# Electrochemical Aptasensor Based on Au Nanoparticles Decorated Porous Carbon Derived from Metal-Organic Frameworks for Ultrasensitive Detection of Chloramphenicol

**DOI:** 10.3390/molecules27206842

**Published:** 2022-10-12

**Authors:** Jing Yang, Jiamin Zou, Wei Zhong, Jin Zou, Yansha Gao, Shuwu Liu, Songbai Zhang, Limin Lu

**Affiliations:** 1 Hunan Provincial Key Laboratory of Water Treatment Functional Materials, Hunan Province Engineering Research Center of Electroplating Wastewater Reuse Technology, College of Chemistry and Materials Engineering, Hunan University of Arts and Science, Changde 415000, China; 2 Key Laboratory of Chemical Utilization of Plant Resources of Nanchang, College of Science, Jiangxi Agricultural University, Nanchang 330045, China

**Keywords:** electrochemical aptasensor, metal-organic framework, gold nanoparticles (Au NPs), chloramphenicol

## Abstract

A facile and sensitive electrochemical aptamer sensor (aptasensor) based on Au nanoparticles-decorated porous carbon (AuNPs/PC) composite was developed for the efficient determination of the antibiotic drug chloramphenicol (CAP). AuNPs modified metal-organic framework (AuNPs/ZIF-8) is applied as a precursor to synthesize the porous carbon with homogeneous AuNPs distribution through a direct carbonization step under nitrogen atmosphere. The as-synthesized AuNPs/PC exhibits high surface area and improved conductivity. Moreover, the loading AuNPs could enhance the attachment of the aptamers on the surface of electrode through the Au–S bond. When added to CAP, poorly conductive aptamer-CAP complexes are formed on the sensor surface, which increases the hindrance to electron transfer resulting in a decrease in electrochemical signal. Based on this mechanism, the developed CAP aptasensor represents a wide linear detection range of 0.1 pM to 100 nM with a low detection limit of 0.03 pM (S/N = 3). In addition, the proposed aptasensor was employed for the analysis of CAP in honey samples and provided satisfactory recovery.

## 1. Introduction

Chloramphenicol (CAP), as a broad-spectrum antibiotic, is commonly applied to treat bacterial infections [1]. Nevertheless, the abuse of CAP can enter the food chain easily and accumulate in the human body [2], resulting in serious bone marrow and blood diseases, such as bone marrow suppression, aplastic anemia, agranulocytosis, and grey baby syndrome etc. [3,4]. Owing to these side effects, CAP usage has been strictly forbidden in many countries such as the United States, Canada, and China [5]. Meanwhile, the European Union (EU) has established a maximum concentration of CAP of 0.3 μg kg^−1^ in animal-origin products [6]. Unfortunately, CAP is used illegally for food-producing animals because of its low cost and high benefits [7]. Therefore, it is critically vital to develop a rapid and simple method for monitoring CAP residues.

To date, several methods, including enzyme-linked immunosorbent assay (ELISA) [8], liquid chromatography–mass spectrometry [9], microfluidic immunoassays [10], and the electrochemical method [11,12] have been developed for CAP detection. In comparison, electrochemical aptamer-based sensors (aptasensors) employing structure-switching aptamers represent a promising platform for the sensitivity and selectivity quantification of target analytes [13]. Aptamers are artificial synthetic oligonucleotide sequences that can bind with a variety of specific receptors such as small organic molecules, heavy metal ions, viruses, proteins, and cells due to the specific binding to their corresponding target molecules with high affinity [14]. Moreover, owing to their relatively easy synthesis and modification, good chemical stability, and high affinity, aptamers have become an important mean of molecular recognition to construct electrochemical aptasensors [15]. For aptasensors, the modified electrode materials play a key role in sensing performance [16]. Therefore, designing appropriate nanomaterials to modify the aptasensor is an effective and useful approach to improve detection performance [17].

Porous carbon materials with large surface areas can be a promising material for electrochemical sensing because of their attractive characteristics, including good stability, excellent interior space for reactant transport, and mass productivity [18]. Among them, metal-organic frameworks (MOFs), which formed by the self-assembly of organic ligands with inorganic building blocks [19], have been used as promising candidates for the effective synthesis of functional porous carbon materials through inert-carbonization methods. Direct carbonization of MOFs by calcination is a common method to improve the conductivity of porous carbon materials. To a certain extent, the resulting carbon materials can preserve the initial attractive structural features of the parent MOFs, such as surface permeability and open-framework structure, which can confer enhanced charge transfer capacity, thus enabling highly improved catalytic performance and conductivity [20]. Thus, MOFs-derived carbons are important electrode modified materials for the development of electrochemical sensors.

Inspired by these characteristics, we demonstrated a well-designed support material based on zeolitic imidazolate framework-8 (ZIF-8)-derived porous carbon-decorated gold nanoparticles (AuNPs/PC) for the detection of CAP. ZIF-8 is a well-known MOF material prepared from zinc ions and 2-methylimidazole, which has been extensively studied in the catalysis fields due to its intersecting three-dimensional structural feature, large pore size, large surface area, and easy preparation, which acts as a good substrate for burdening AuNPs and preventing the aggregation of AuNPs [21]. The AuNPs/PC composite was then obtained by an inert carbonization process using AuNPs/ZIF-8 composite as starting material, where the AuNPs were deposited on the porous carbon matrix to improve the biocompatibility of the surface for biomolecules. The sulfhydryl group (-SH) modified aptamer was immobilized on the electrode surface through Au-S bond [22]. In the presence of CAP, the formed CAP-aptamer complexes increase the hindrance of electron transfer, resulting in a decrease of the current signal. The proposed electrochemical aptasensor demonstrates high selectivity and sensitivity for the CAP detection and is successfully applied to real samples with a satisfactory result.

## 2. Results and Discussion

### 2.1. Characterization of Composites

**Figure 1:**
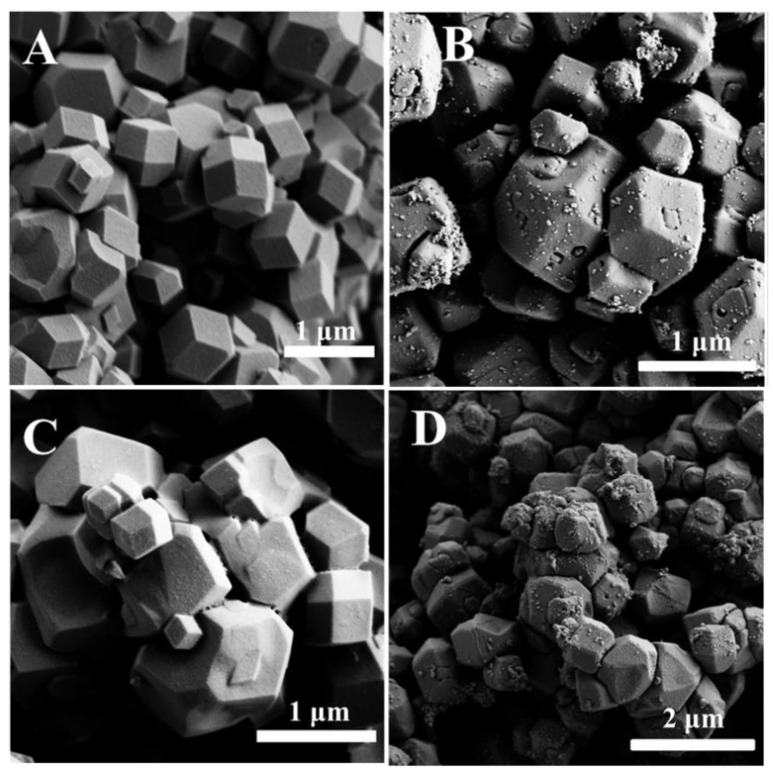
The SEM images of (**A**) ZIF-8, (**B**) AuNPs/ZIF-8, (**C**) PCs, and (**D**) AuNPs/PCs.

The morphologies of ZIF-8, Au/ZIF-8, PCs, and Au/PCs were evaluated by SEM. As illustrated in Figure 1A, ZIF-8 shows a classic rhombic dodecahedron structure with smooth surfaces and uniform size distributions, and the average diameter of the formed ZIF-8 is about 800 nm. The metal precursor diffuses into the surface and pores of the prefabricated highly crystalline ZIF-8 and is directly reduced in situ to form small Au nanoparticles (Figure 1B). These methods avoid the aggregation of nanoparticles and retain the inherent crystallinity and nano porosity of the MOF used. After a pyrolysis process, the skeleton of ZIF-8 is transferred in situ into PCs, and the obtained PCs (Figure 1C) and AuNPs/PCs (Figure 1D) basically maintain the dodecahedron morphology of MOFs precursors.

**Figure 2:**
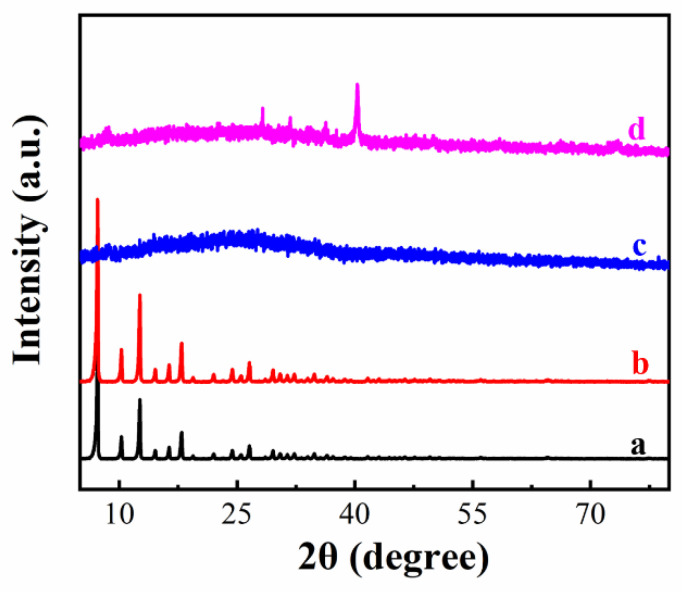
XRD patterns of ZIF-8 (**a**), AuNPs/ZIF-8 (**b**), PCs (**c**), and AuNPs/PCs (**d**).

Figure 2 demonstrates the XRD patterns of ZIF-8, AuNPs/ZIF-8, PCs, and AuNPs/PCs powders. The sharp diffraction peaks of synthesized ZIF-8 crystals (curve a) indicate the excellent crystallinity, with the major diffraction peaks at 7.43°, 10.45°, 12.68°, 14.75°, 16.45°, 18.02°, 19.45°, and 22.21°, which is consistent with those previously reported [23]. For AuNPs/ZIF-8, two characteristic peaks of gold appear at 38.18° and 44.4° and perfectly matched the (111) and (200) faces of Au crystal. After loading Au into ZIF-8, there is no significant loss of the crystallinity, suggesting that the integrity of ZIF-8 is maintained in the AuNPs/ZIF-8 composite. After heating, there are two distinct peaks at 25° and 44° for PCs (curve c), attributing to the carbon (002) plane and (101) plane, which are considered to be amorphous carbon [24]. Furthermore, for AuNPs/PCs, the spectrum is well matched with those of PCs and Au, which indicates that the AuNPs/PCs composite was successfully synthesized.

### 2.2. Electrochemical Characterizations

To probe whether the feature of the modified electrode is successfully constructed, each step of the CAP aptasensor was monitored using cyclic voltammetry (CV) and electrochemical impedance spectroscopy (EIS) techniques in the 5 mM [Fe(CN)_6_]^3−/4−^ mixture (1:1) with 0.1 M KCl solution.

**Figure 3:**
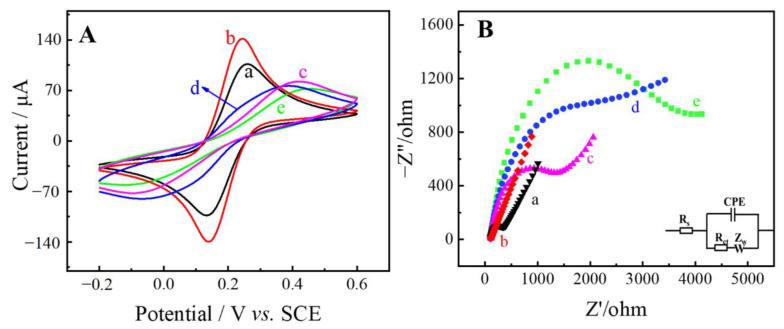
The Cyclic voltammetry (**A**) and Electrochemical impedance spectroscopy (**B**) characterization of the construction process of CAP electrochemical aptasensor (a. bare GCE, b. AuNPs/PCs/GCE, c. Apt/AuNPs/PCs/GCE, d. MCH/Apt/AuNPs/PCs/GCE and e. CAP/MCH/Apt/AuNPs/ PCs/GCE) in 5 mM K_3_Fe(CN)_6_/K_4_Fe(CN)_6_ (1:1) solution containing 0.1 M KCl.

As can be seen in Figure 3A, the bare electrode (curve a, bare GCE) exhibits a well-defined pair of reversible redox peaks. Compared to the bare GCE, a significantly increased reversible redox peak current and a decrease in peak potential separation (ΔE*_p_*) are observed after the immobilization of AuNPs/PCs on GCE (curve b, AuNPs/PCs/GCE). This is due to the fact that the composite of AuNPs/PCs could improve the conductivity of the electrodes effectively. After the immobilization of aptamer (curve c, Apt/AuNPs/PCs/GCE), an obvious decrease in peak current is found. It could be explained by the negatively charged phosphate groups in the aptamer backbone, which obstructs the negatively charged redox probe [Fe(CN)_6_]^3−/4−^ from reaching electrode surface [25]. Subsequently, when MCH is added to block nonspecific sites, the peak current shows a further decrease because of the non-conductive property of MCH (curve d, MCH/Apt/AuNPs/PCs/GCE). Finally, after incubation of the CAP, the peak current drops similarly (curve e, CAP/MCH/Apt/AuNPs/PCs/GCE), indicating the successful capture of CAP by the aptamers. The above results indicate that the construction of the electrochemical aptasensor was successful.

EIS was also used to track the interfacial interactions of the aptasensor. As depicted in Figure 3B, the Nyquist diagram consists of a semicircular part and a linear part. As is well known, the semicircular in the high-frequency region corresponds to the charge transfer resistance (R_ct_), which can be estimated from the diameter of the semicircle. The semicircle diameter of bare GCE is relatively large (curve a, 320.7 Ω cm^2^). When AuNPs/PCs is doped on the electrode (curve b, 105.4 Ω cm^2^), the semicircle diameter decreases significantly, which indicates that AuNPs/PC is successfully modified onto the electrode. With the modification of thiol-modified aptamers onto the AuNPs/PCs/GCE surface, the semicircle diameter of Apt/AuNPs/PCs/GCE (curve c, 1513 Ω cm^2^) increase, which is owing to the effect of electrostatic repulsion between [Fe(CN)_6_]^3−/4−^ and the negatively charged phosphate groups in aptamer [26]. Next, the assemble of MCH causes a further increase of the semicircle diameter owing to its insulating property (curve d, 2822 Ω cm^2^). Finally, with the capture of CAP by the aptamers, the R_ct_ increases to 3555 Ω cm^2^. Both the results from the cyclic voltammetric and EIS indicate the successful construction of the designed electrochemical aptasensor.

### 2.3. Optimization of Experimental Conditions

To get the excellent analytical performance of CAP-aptasensor, some crucial experimental conditions were optimized, namely the volume of AuNPs/PCs, the concentration and incubation time of aptamer, and the reaction time of CAP. The inhibition ratio is utilized to evaluate the constructed aptasensor, which is calculated as follows:

ΔI/I_0_ (%) = [(I_0_ − I_1_)/I_0_] × 100 (1)

where I_0_ and I_1_ delegate the current of the aptasensor before and after incubation with CAP.

**Figure 4:**
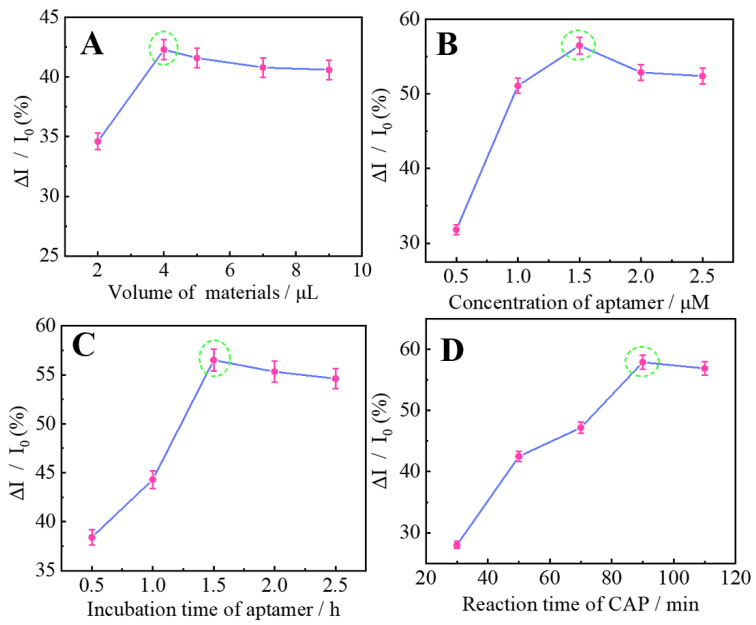
The optimization of (**A**) the volume of AuNPs/PCs, (**B**) the concentration of aptamer, (**C**) the incubation time of aptamer, and (**D**) the incubation time of CAP.

To investigate the effect of the volume of AuNPs/PCs on the aptasensor, different volumes (2.0–9.0 μL) were studied under the experimental conditions of 1.5 µM concentration of aptamer, 1.5 h incubation time of aptamer, and 90 min reaction time of CAP. As demonstrated in Figure 4A, the inhibition ratio increases as the increasing volume of AuNPs/PCs ranges from 2.0 to 4.0 μL and reaches the maximum value at 4.0 μL. This phenomenon might be due to excessive AuNPs/PCs built upon the electrode surface, hampering electron transfer. Hence, the optimal volume of AuNPs/PCs is confirmed to be 4.0 μL.

The aptamer concentration and incubation time were directly associated with the aptasensor signal. To determine the optimal incubation time, the effect of different incubation times (0.5–2.5 μM) was investigated under the experimental conditions of 4.0 μL volume of AuNPs/PCs, 1.5 h incubation time of aptamer, and 90 min reaction time of CAP. As indicated in Figure 4B, the inhibition ratio increases with the increase of the aptamer concentration ranging from 0.5 to 1.5 μM. However, the inhibition ratio decreases when further increases the concentration. This indicates that the active sites on the surface of AuNPs/PCs are completely saturated when the aptamer concentration reaches 1.5 μM. However, the inhibition ratio decreases gradually at higher concentration of aptamer (>1.5 μM), indicating that over-saturation of the aptamer on the electrode surface could impede the electron transfer between the electrode surface and the signal probe. The incubation time is also an important element affecting the sensing performance of the aptasensor. Experimental results for different aptamer incubation times (0.5, 1.0, 1.5, 2.0 and 2.5 h) under the experimental conditions of 4.0 μL volume of AuNPs/PCs, 1.5 µM concentration of aptamer, and 90 min reaction time of CAP were shown in Figure 4C. The inhibition ratio increases sharply with the increased immobilization time from 0.5 to 1.5 h, and tends to become a stabilized platform after 1.5 h, indicating that the aptamer is saturated on the surface of GCE. Therefore, 1.5 μM and 1.5 h are chosen as the optimal aptamer concentration and incubation time.

CAP and aptamer exist in a specific combination, which is associated the inhibition ratio of sensor. Therefore, the incubation time between Apt and the target is also an important parameter that influences the analytical performance of the aptasensor. Figure 4D displays the effect of different CAP incubation times (30, 50, 70, 90 and 110 min) on the aptasensor under the experimental conditions of 4.0 μL volume of AuNPs/PCs, 1.5 µM concentration of aptamer, and 1.5 µM concentration of aptamer. It could be observed that the inhibition ratio increases gradually as the incubation time increases from 30 to 90 min and remains stable after 90 min. This result demonstrates that the binding between Apt and CAP has reached saturation. Thus, 90 min is chosen for the optimum reaction time of CAP.

### 2.4. Analytical Performance of the Aptasensor

**Figure 5:**
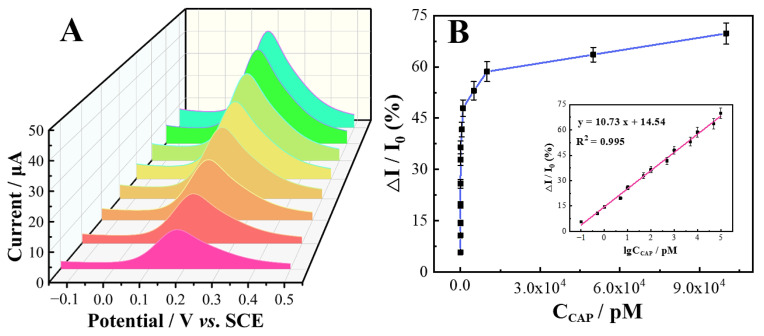
(**A**) DPVs of the aptasensor in different concentration of CAP in 5 mM [Fe(CN)_6_]^3−/4−^ (1:1) containing 0.1 M KCl (from 0.1, 1, 10, 100, 1 × 10^3^, 1 × 10^4^, 1 × 10^5^ pM). (**B**) Dependence of inhibition ratio on the concentration of CAP. Insert shows the linger curve of ΔI/I_0_ vs. lgC.

Under the optimum conditions, the aptasensor based on the MCH/Apt/AuNPs/PCs/GCE sensing platform was applied to quantitatively detect CAP with DPV measurements. Figure 5A shows the DPV curve of CAP-aptasensor to different concentrations (0 pM~100 nM). The relationship between the inhibition ratio and CAP concentration is depicted in Figure 5B. The inhibition ratio gradually decreases with the increase of CAP concentration. The inhibition ratio is linearly correlated with the logarithm of CAP concentration ranging from 0.1 pM to 100 nM, with the linear equation of ΔI/I_0_ = 10.73 lgC_CAP_ + 14.54 (R^2^ = 0.995). The LOD of 0.03 pM is calculated based on S/N = 3.

Furthermore, the analytical performance of Apt/AuNPs/PCs/GCE is compared with previously reported electrochemical sensors for detecting CAP (Table 1). By comparison, the aptasensor in this work displays a remarkable analytical property with both a wider linear range and lower LOD for CAP.

### 2.5. Selectivity Investigation of Aptasensor

**Figure 6:**
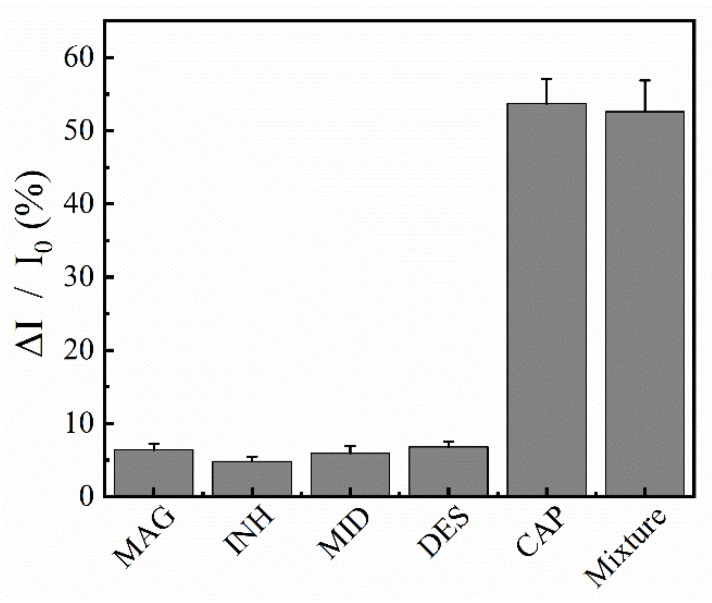
Selectivity studies of aptasensor in existing of 10 nM CAP and 500 nM magnolol (MAG), isoniazid (INH), midecamycin (MID), and diethylstilbestrol (DES).

Good selectivity of the sensor towards CAP detection in the presence of identical species is an important parameter in its assessment. Some antibiotics including diethylstilbestrol (DES), midecamycin (MID), isoniazid (INH), and magnolol (MAG), which have similar functions with CAP and were chosen as potential interferents to investigate the selectivity of the developed aptasensor. As can be seen in Figure 6, all of these interferents (500 nM) show little effect on the inhibition ratio of the aptasensor, while CAP shows a favorable ratio of inhibition, although the concentration of CAP (10 nM) is much lower than that of the other antibiotics. Moreover, the mixed solution containing all the above antibiotics and pure CAP shows little change in ratio of inhibition, reflecting the high binding specificity of the CAP aptamer to the CAP target. All the results exhibit that the design aptasensor has a good selectivity to CAP detection.

### 2.6. Real Sample Analysis

The feasibility of the proposed aptasensor was assessed by the standard addition/recovery method using honey as real samples. The honey samples were obtained from supermarket. Before determination, the honey was diluted ten times with 0.1 M PBS, and the mixture was then centrifuged at 10,000 rpm for 10 min to obtain the supernatant. Finally, a recovery test was carried out by adding different concentration (100, 500, and 1000 pM) of CAP into these obtained supernatants, and the recoveries obtained are summarized in Table 2. As shown, the recovery ranges from 95.4% to 103.0% (*n* = 3), and all the RSDs are less than 5%. Moreover, the results are in good agreement with the inductively coupled plasma mass spectrometry (ICPMS) method. These results indicate that the prepared aptasensor based on AuNPs/PCs is reliable for detecting CAP in real sample.

## 3. Materials and Methods

### 3.1. Reagents

2-methylimidazole (C_4_H_6_N_2_), Zinc nitrate hexahydrate (Zn(NO_3_)_2_·6H_2_O), gold (III) chloride tetrahydrate (HAuCl_4_·4H_2_O, 99%), KCl, K_4_[Fe(CN)_6_], and K_3_[Fe(CN)_6_] were purchased from Ganyi Reagent Co. Ltd. (Nanchang, China). Chloramphenicol (CAP), 6-Mercapto-1-hexanol (MCH), diethylstilbestrol (DES), isoniazid (INH), midecamycin (MID), and Magnolol (MAG) were acquired from Yuanye Bio-Technology Co., Ltd. (Shanghai, China). Ultrapure water was available from the system of Milli-Q water purification. Phosphate buffered saline (PBS, 0.1 M, pH 7.4) was formulated with Na_2_HPO_4_, NaH_2_PO_4_ and KCl solutions, which was used to dissolve the [Fe(CN)_6_]^3−/4−^ signal probe used in the subsequent measurements. MCH was diluted to 1 mM with Tris-HCl buffer.

The oligonucleotide was provided and purified by Sangong Bio-technology (Shanghai, China) Co., Ltd., and the base sequence was followed. CAP-binding aptamer sequence [29]: 5′-C6/SH-ACT TCA GTG AGT TGT CCC ACG GTCGGC GAG TCG GTG GTA G-3′.

### 3.2. Apparatus

All electro-analytical experiments were performed on a CHI-750E electrochemical workstation (Shanghai CH Instrument Corporation, Shanghai, China) using a common three-electrode system, which comprised a Pt wire electrode, saturated calomel electrode (SCE), and glass carbon electrode (GCE, *Ф* = 3 mm). For characterization, SEM (TESCAN MIRA, Shanghai, China) and XRD (Rigaku Corporation diffractometer, Tokyo, Japan) were used.

### 3.3. Synthesis of AuNPs/ZIF-8

The ZIF-8 was prepared with slight modifications based on the previously reported method [32]. In total, 0.6 g of Zn(NO_3_)_2_·6H_2_O was dissolved in 40 mL of CH_3_OH by ultrasonic dispersion. Afterward, the above suspension was slowly added to 40 mL of CH_3_OH solution containing 2.6 g of 2-methylimidazole under vigorous stirring for 1 h and settled down for 24 h. The obtained nanoparticles were collected by centrifugation and washed with methanol 3 times, then dried at 60 °C overnight.

To synthesize the AuNPs/ZIF-8 [18], the prepared ZIF-8 (0.2 g) was dissolved in 25 mL ethanol-water (*v*/*v*: 1/1) solution under the help of ultrasound. Then, 0.25 mL of HAuCl_4_ (20 mg·mL^−1^) was added to the above solution under stirring. Afterward, 2.5 mL of NaBH_4_ (0.05 M) was added and mixed for 4 h. The whole process was finished in an ice bath. The AuNPs/ZIF-8 was obtained by centrifugation, and dried overnight in a vacuum drying oven.

### 3.4. Synthesis of AuNPs/PCs

The AuNPs/PCs was prepared based on a facile carbonization process, whereby 0.2 g AuNPs/ZIF-8 was heated to 800 °C at 3 °C min^−1^ under a nitrogen environment and held 800 °C for 2 h [33].

### 3.5. Fabrication of the Aptasensor

**Scheme 1:**
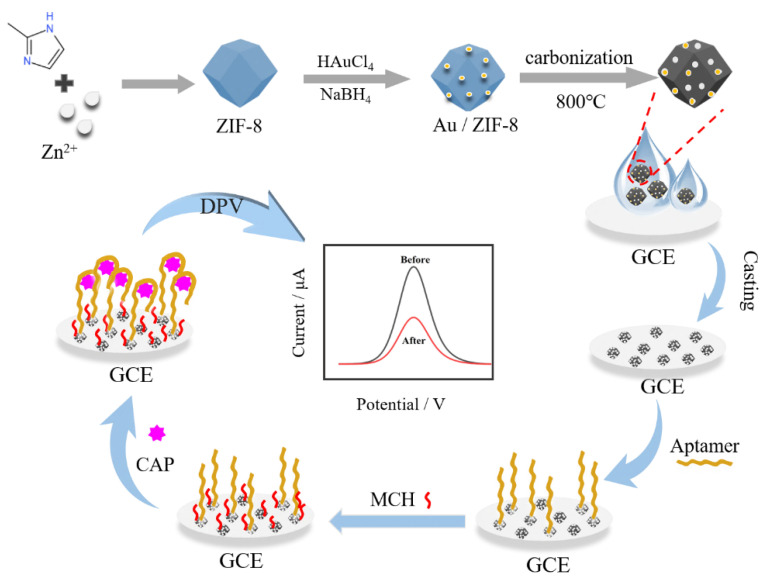
Schematic diagrams of fabricating the designed electrochemical aptasensor based on AuNPs/PCs for CAP detection.

A total of 5 mg of AuNPs/PCs was dispersed into 1 mL DMF to obtain a well-dispersed suspension under ultrasound. Before modification, the surface of GCE was polished with Al_2_O_3_ powder for 5 min, followed by ultrasonic washing with ultrapure water and ethanol in sequence. Then, 5 μL AuNPs/PCs suspension was pipetted on the surface of GCE and dried under the infrared lamp to obtain AuNPs/PCs/GCE. Then, the AuNPs/PCs/GCE was incubated with 10 μL aptamer (1 μM) at 37 °C for 1.5 h, followed by washing with ultrapure water to remove the unbounded aptamer. Afterward, the MCH (1 mM, 5 μL) solution was applied on electrode surface for 15 min for blocking the non-specific binding sites and subsequently cleaned by ultrapure water. The resulting aptasensor was placed in a refrigerator. The fabrication of the aptasensor is presented in Scheme 1.

### 3.6. Electrochemical Measurements

Electrochemical impedance spectroscopy (EIS) measurements were performed in the 0.1 M KCl solution containing 5.0 mM [Fe(CN)_6_]^3−/4^^−^ with a frequency from 0. 1 Hz to 10^4^ Hz. Differential pulse voltammetry (DPV) measurements were recorded in 5.0 mM [Fe(CN)_6_]^3−/4−^ solution containing 0.1 M KCl in the potential range of −0.2–0.6 V. Cyclic voltammetry (CV) measurements (potential range of −0.2–0.6 V, scan rate of 100 mV s^−1^) were obtained to test the performance of the prepared electrodes.

## 4. Conclusions

In conclusion, a novel and sensitive electrochemical aptasensor was developed for the highly effective detection of trace CAP based on the AuNPs/PCs/GCE. AuNPs/PCs are directly synthesized by a simple one-step carbonization of AuNPs/ZIF-8 at 800 °C without additional precursors. The obtained AuNPs/PCs combine the advantages of PCs and AuNPs including high surface areas, good conductivity, biocompatibility, and abundant pore structure. The proposed aptasensor not only displays a low detection limit, but also owns favorable selectivity and admirable repeatability for detecting CAP. Additionally, satisfactory recoveries are obtained in the determination of CAP in honey samples, indicating that the proposed method shows considerable potential for analytical applications.

## Figures and Tables

**Table 1:** Comparison of analytical performance of various reported electrochemical sensors for CAP detection.

Modified Electrode	Method	Linear Range (nM)	LOD (nM)	Ref.
Apt/rGO/AgNPs/GCE	LSV	10–35 × 10^3^	2	[27]
PEI-C_3_N_4_/AuNWs	DPV	0.1–1000	2.69 × 10^−3^	[28]
Apt/PEI-rGO/AuNCs/AuE	DPV	0.005–1 × 10^3^	2.08 × 10^−3^	[29]
rGO@PDA@AuNPs	DPV	100–100 × 10^3^	58	[30]
Apt/AgNPs/[NH_2_–Si]-f-GO/GCE	DPV	0.01–200	0.0033	[31]
Apt/AuNPs/PCs/GCE	DPV	0.0001–100	0.03 × 10^−3^	This work

Note: PEI: Polyethyleneimine; AuE: Gold Electrode; PDA: Polydopamine; [NH_2_–Si]-f: functionalized with (3-Aminopropyl) triethoxysilane.

**Table 2:** Recovery results of CAP in honey samples (*n* = 3).

Samples	Added (pM)	Found (pM)	RSD (%)	Recovery (%)	by ICPMS Method
1	100	103	2.17	103.0	98.41
2	500	477	2.96	95.4	511.81
3	1000	985	1.88	98.8	1017.59

Note: RSD: relative standard deviation.

## Data Availability

The data presented in this study are available in article.

## Sample Availability

Samples of the compounds are available from the authors.
